# Effects of yeast culture supplementation on milk yield, rumen fermentation, metabolism, and bacterial composition in dairy goats

**DOI:** 10.3389/fvets.2024.1447238

**Published:** 2024-08-07

**Authors:** Zunyan Li, Yufeng Hu, Haibin Li, Yingting Lin, Ming Cheng, Fenghua Zhu, Yixuan Guo

**Affiliations:** ^1^College of Animal Science and Technology, Qingdao Agricultural University, Qingdao, China; ^2^Qingdao Animal Husbandry and Veterinary Research Institute, Qingdao, China

**Keywords:** dairy goats, production performance, rumen fermentation parameters, rumen microflora, rumen metabolism

## Abstract

The effects of yeast culture (YC) on dairy goat milk yield and potential effects of rumen microbial population changes on rumen fermentation are poorly understood. This study aimed to evaluate the effects of YC on milk yield and rumen fermentation in dairy goats and explore the potential microbial mechanisms. Forty Laoshan dairy goats with a weight of 51.23 ± 2.23 kg and daily milk yield of 1.41 ± 0.26 kg were randomly divided into 4 groups: control (no YC), YC1 (10 g/day per goat), YC2 (25 g/day per goat), and YC3 (40 g/day per goat). The pre-feeding period was 15 days, and the official period was 60 days. Laoshan dairy goats were milked twice daily, and the individual milk yield was recorded. On the last day of the official period, rumen fluid was collected to measure rumen fermentation, perform quantitative polymerase chain reaction (PCR), and detect metabolites. Compared to the control group, the YC group had greater milk yield; higher acetic acid, butyric acid, and total volatile fatty acid contents; and lower ammonia-N (NH_3_-N) content in the rumen (*p* < 0.05). YC increased the abundance of *Clostridia_UCG-014* and *Paraprevotella* (*p* < 0.05). Differential metabolites L-leucine and aspartic acid were screened. This study revealed the microbial mechanisms linking the relative abundance of *Paraprevotella* and *Clostridia_UCG-014* to L-leucine and aspartic acid utilization. These results describe the potential benefits of supplementing 10 g/day per goat YC in the diets of Laoshan dairy goats for improving the rumen environment and milk yield.

## 1 Introduction

Increasing dairy production efficiency is a key goal in dairy goat nutrition, and dietary interventions play a crucial role. Yeast culture (YC) has been reported to improve milk yield (1, 2), stabilize rumen pH, and promote consistent environments for rumen fermentation (3–5).

Commercial YC and yeast-containing feed ingredients vary in many characteristics, including the yeast strain, viability, culture and associated media, and post-fermentation processing [e.g., fractionated yeast (6)]. YC is unique among yeast products because it contains yeast biomass and fermentation metabolites (6). The composition and characteristics of the fermentation metabolites are highly dependent on the medium used to grow the yeast. The freeze-drying method of yeast culture can also retain a small amount of active yeast and the fermentation activity of yeast (6, 7). The benefits of YC have been attributed to the presence of functional metabolites (e.g., organic acids, B vitamins, and enzymes) that may influence ruminal fermentation by supplying key nutrients that are otherwise scarce in the ruminal environment (8, 9).

The abundance of various rumen bacterial taxa is correlated with production performance and rumen fermentation parameters, indicating that bacterial communities play important roles in regulating host physiological parameters (10, 11). According to Chaucheyras-Durand et al. (12), the main effects of YC are related to rumen fermentation and benefit key microbial populations and their metabolism, increase fiber degradation, and stabilize rumen pH. *In vivo* and *in vitro* experiments have shown that YC can stimulate the growth of rumen cellulolytic bacteria (13), promote fiber degrading bacteria establishment in the digestive tract of lambs, and accelerate microbial activity in the rumen (14). Rumen cellulolytic bacteria can decompose dietary macromolecular carbohydrates into glucose, which is then fermented to produce volatile fatty acids (VFAs), such as acetic acid, propionic acid, and butyric acid. These VFAs contain large amounts of energy and are the main energy sources for dairy cows (15, 16). In addition, studies have reported that the ratios of these VFAs are affected by changes in microbial metabolism and species (17). However, the effects of YC on the production performance and rumen fermentation parameters of dairy goats and the exact microbial mechanisms underlying these effects are unclear. Therefore, understanding the microbial mechanisms underlying the effects of YC on the rumen is important for optimizing the utilization of YC for ruminant nutrition.

Therefore, the current study evaluated the effects of YC on the production performance, rumen fermentation characteristics, rumen microorganisms, and metabolites of dairy goats. Moreover, the main metabolites and metabolic pathways affecting rumen fermentation parameters were explored through the weighted gene correlation network analysis (WGCNA) method to further understand the microbial mechanisms underlying the effect of YC on rumen fermentation parameters in dairy goats.

## 2 Materials and methods

### 2.1 Ethical considerations and location

This study was conducted at the Aote Breeder Goat Co., Ltd., Qingdao City, Shandong Province (120°36′E, 36°58′N), and the experiments were performed in strict accordance with the guidance of the National Council for the Control of Animal Experimentation under an experimental protocol approved by the Animal Science and Technology College of Qingdao Agricultural University, Qingdao, Shandong (protocol code DKY20200701, dated July 1, 2020).

### 2.2 Diet and livestock management

The studied YC, which was provided by Danongwei Technology (Shenzhen) Co., Ltd., is a concentrated and dried product of *Saccharomyces cerevisiae* after fermentation. It is mainly composed of yeast cell lysate, yeast cell wall (e.g., mannan-oligosaccharide, β-glucan, and chitin), post-fermentation denaturation medium, and extracellular metabolites (e.g., nucleosides, organic acids, proteins, peptides, plant Zi alcohol, natural antioxidants, and digestive enzymes). The crude protein (CP) content was 16.2%, crude fat (EE) content was 1.53%, ash content was 7.71%, and moisture content was 9.87%.

A completely randomized experimental design was adopted. Forty Laoshan adult dairy goats (born in late April 2020) that had previously given birth twice and exhibited good body condition, (51.23 ± 2.23 kg body weight; 40 ± 4 days in milk; 1.41 ± 0.26 kg/d milk yield) were selected and randomly divided into four groups. Each treatment consisted of 10 Laoshan dairy goats.

The diets were formulated based on the nutrient requirements of lactating goats reported by Cannas et al. (18, 19). The goats were fed a basal diet *ad libitum* and allowed approximately 5% orts twice a day at 08:00 and 14:30. The daily feeding quantity was 3.5% of the body weight. The dietary composition and nutritional levels are shown in Table 1.

**Table 1:** Ingredients and chemical composition of diets (DM basis).

Item	g/100 g
Ingredients	
Corn silage	16.46
Peanut vine	20.77
Pomace	6.21
Garlic stalks	3.53
Hay	2.16
NaCl	0.29
Mineral-vitamin-protein mix^1^	50.58
Total	100
Nutrient content^2^, dry matter basis	
DE, MJ/kg	13.85
CP, %	13.95
NDF, %	47.59
ADF, %	22.19

1 Mineral-vitamin-protein mix was purchased from Oulifeide Feed Science and Technology Co, Ltd. (Shandong, China), and it contained corn, corn dry alcohol grains, spray corn husk, corn germ meal, soybean meal, bran, cotton meal, calcium bicarbonate, stone powder, sodium chloride, sugarcane molasses, palm fat powder, sodium bicarbonate, minerals, and vitamin ingredients. The feed had the following contents, crude protein (CP), 20.1%; Ash, 9.7% and NaCl, 1%.

2 For the nutrient levels, the digestible energy (DE) was based on the calculated value. CP, neutral detergent fiber (NDF), and acid detergent fiber (ADF) were the measured value.

The control group (CON) was fed a basic diet, while the YC1, YC2, and YC3 groups were fed the basic diet and 10, 25, and 40 g YC/day per goat, respectively. To ensure that the YC groups ingested sufficient YC per day per goat, YC was mixed with a small amount of basic diet and fed to each goat separately every morning. After each goat had finished all of the basic diet with YC, the remaining feed would be given to them. The YC dose was based on the recommendations of the supplier, which suggested a range of 10–40 g/day per goat. The adaptation period was 15 days, and the experimental period was 60 days. During the test period, all Laoshan dairy goats were allocated to individual pens with natural light and cool environmental temperatures and provided *ad libitum* access to drinking water.

### 2.3 Data and sample collection

Laoshan dairy goats were milked twice daily at approximately 06:00 and 18:00, and individual milk yield was recorded using an Afikim milking system (AfiFlo milk meters, S.A.E. Afikim, Israel). The milk samples (morning and afternoon) of each animal were mixed to form a composite sample, which was placed in plastic containers with Brono-pol^®^ preservative and stored in a freezer at −20°C until the chemical composition (50 mL sample) was analyzed. Milk fat concentrations were analyzed once weekly using the Gerber method (20). The 4% fat corrected milk (FCM) yield was calculated as follows: [(0.4 × milk yield) + (15 × milk fat percentage × milk yield)] (21).

The rumen fluid was collected from 40 Laoshan dairy goats on the final day of the study period. Laoshan dairy goats were sampled in the afternoon via an esophageal tube at 14:00. The esophageal tubing apparatus was assembled by coupling the esophageal tube to a metal strainer (22) at one end and the opposite handle side of a manual vacuum pump (Med-Eze stomach pump, MAI Animal Health, China) at the other end. The rumen fluid samples were collected by passing the fluid through the hollow shaft of the pump into a plastic beaker. After discarding the first 200 mL of fluid to minimize salivary contamination, approximately 50 mL of rumen fluid was collected. After collection, the pH was immediately measured using a pH meter (Waterproof pH Testr 30, Oakton Instruments, United States), and two aliquots (10 mL) were acidified with either 200 μL of 50% sulfuric acid or 2 mL of 25% meta-phosphoric acid and stored at −20°C until analysis of ammonia-N (NH_3_-N) and VFAs, respectively. In addition, 2 mL of rumen fluid samples were collected and immediately frozen in liquid nitrogen and stored at −80°C until DNA isolation and subsequent relative abundance analysis of bacteria species, which was performed via the quantitative polymerase chain reaction (qPCR) method. Detection of metabolites was performed via non-targeted metabolomics analysis on Ultra-high-performance Liquid Chromatography–Tandem Mass Spectrometry (UHPLC–MS/MS) system (ExionLC AD, SCIEX, United States; QTRAP^®^, SCIEX, United States).

Rumen fluid samples preserved in 50% sulfuric acid and 25% meta-phosphoric acid were thawed and transferred into 2 mL microcentrifuge tubes. Then, the samples were centrifuged at 30,000 × *g* for 20 min at 4°C (model 5403, Eppendorf, Germany), and the supernatant from samples in sulfuric acid was used to analyze NH_3_-N using the colorimetric assay described by Chaney and Marbach (23). The supernatant of rumen fluid containing 25% meta-phosphoric acid was analyzed for the acetic acid, propionic acid, butyric acid, and total VFA (TVFA) concentrations using an automated gas chromatograph (model 689, Hewlett-Packard, Juyi Hui supply chain Co., Ltd., China) equipped with a 0.25 mm i.d × 15-m column (Nukol 24106-U, Supelco, Inc., United States), and the internal standard was 2-ethylbutyrate.

### 2.4 Ruminal bacteria DNA isolation and qPCR amplification of 16S rDNA genes

Microbiome DNA was extracted from each sample (250 μL of rumen fluid was used) using a PowerSoil DNA Isolation Kit (MoBio Laboratories, Inc., Canada), following the manufacturer’s instructions. The extracted DNA was detected using a NanoDrop 2000 (ThermoFisher Scientific, Inc., United States) to determine the DNA quality and concentration. The samples qualified for quality inspection were stored at −20°C for use in follow-up experiments.

The V3-V4 region of the 16Sr RNA gene of bacteria was amplified by primers 338F (5′-ACTCCTACGGGAGGCAGCAG-3′) and 806R (5′-GGACTACHVGGGTWTCTAAT-3′). Eight base-pair barcode sequences were added to the 5′ ends of upstream and down-stream primers to distinguish different samples. The PCR reaction system contained the following (25 μL total volume): 12.5 μL 2× Taq Plus Master Mix II (Vazyme Biotech Co., Ltd., China), 3 μL BSA (2 ng/μL), 1 μL forward primer (5 μM), 1 μL reverse [rimer (5 μM), 2 μL DNA (total amount of added DNA was 30 ng)], and 5.5 μL dd H_2_O to a volume of 25 μL. The reaction parameters were pre-denatured at 95°C for 45 min, denatured at 95°C for 45 s, annealed at 55°C for 50 s, annealed at 55°C for 45 s, extended at 72°C for 45 s for 28 cycles, and extended at 72°C for 10 min. The PCR products were amplified using an ABI 9700 PCR instrument (Thermo Fisher Scientific, Inc., United States). The size of the amplified bands was detected by 1% agarose gel electrophoresis, and the bands were purified using an Agencourt AMPure XP nucleic acid purification kit (Beckman Coulter, Inc., United States).

The library was constructed using the NEB Next Ultra II DNA Library Prep Kit (New England Biolabs, Inc., United States), which is a library building kit, and paired-end sequencing was performed using the Illumina MiSeq PE300 (Illumina, Inc., United States) high-throughput sequencing platform. Trimmomatic software was used to control the quality of Fastq data, and it used the sliding window strategy, a window size of 50 mbp, average quality value of 20, and minimum reserved sequence length of 120. Using Pear (v0.9.6), the minimum overlap was set to 10 bp, and the mismatch rate was 0.1. After splicing, Vsearch (v2.7.1) software was used to remove sequences whose length was less than 230 bp, and the UCHIME method was used to remove chimera sequences according to the Gold Database. Using the Vsearch (v2.7.1) software uparse algorithm for operational taxonomic unit (OTU) clustering of high-quality sequences. Valid tags with sequence similarity thresholds ≥97% were assigned to the same taxon (OTU), and the tag sequence with the highest abundance was selected as the representative sequence in each OTU cluster. The BLAST algorithm with Silva138 was used to annotate the species classification. The α diversity index results were analyzed by QIIME (v2.0.0) software, and the α diversity among groups was compared by Wilcoxon rank test using the R package ggpubr (0.4.0). Based on the species annotation and relative abundance results, R (v3.6.0) software was used to analyze the histogram of species composition. QIIME (v2.0.0) was used to calculate the beta diversity distance matrix. NMDS analysis and mapping were performed using the RPG plot2 (3.3.2) and vegan software packages. The Sankey diagram of the species community composition was visualized using the R-package ggplot2. Short time-series expression miner (STEM) (24). The abundance distributions of all OTUs were analyzed. Co-occurrence network analysis was performed using the R-package Psych and visualized using the Cytoscape software (version 3.7.1).

### 2.5 Metabolite extraction and UHPLC–MS/MS analysis

Rumen fluid samples were collected in 1.5 mL Eppendorf miniature centrifuge tubes, and 1 mL 70% methanol internal standard extract was added. The samples were oscillated for 5 min, maintained on ice for 15 min, centrifuged at 12000 × *g* for 10 min at 4°C. The supernatant was then extracted, and 400 μL was placed in a corresponding EP tube after centrifugation. The supernatant was then placed in a −20°C refrigerator overnight. Then, it was centrifuged at 12000 × *g* for 3 min at 4°C. Subsequently, 200 μL of supernatant was placed into the inner liner of the corresponding injection bottle for UHPLC–MS/MS analysis, which combined ultra-performance liquid chromatography (UPLC) (ExionLC AD, SCIEX, United States) with MS/MS (QTRAP^®^, SCIEX, United States). Samples collected from the supernatant mixture were used as quality control samples. During the instrument analysis, one quality control sample was injected into every six test samples to monitor the repeatability and stability of the instrument.

The original data obtained from the UHPLC–MS/MS platform were converted into the TXT format using MSconventer software. Based on the self-built target database MWDB (software database), the software Analyst1.6.3 was used to qualitatively analyze the information and secondary spectrum data according to the retention time (RT), parent ion pair, and secondary spectrum data. Heat maps of different metabolites were drawn using the R-package pheatmap. Metabolites were enriched using MetaboAnalyst4.0.^1^

The WGCNA method is a correlation-based method that describes and visualizes networks of data points, regardless of whether they are estimates of gene expression, metabolite concentration, or other phenotypic data (25, 26). WGCNA can identify a module by building a metabolite correlation network and deriving the characteristic metabolite score (related to the first principal component) (27) from the identified module. To explore the mechanism of rumen acid-related metabolism, WGCNA was used to identify highly related metabolite modules based on annotated metabolites. These modules were associated with the rumen acid indexes determined in this study. The parameters used were a soft power of five and a minimum module size of 10 metabolites. The modules with *p* < 0.05 and *R* > 0.5 were considered to be related to the rumen acid index. The molecule pathway database MetaboAnalyst4.0^2^ was used to analyze the pathway enrichment of metabolites in the rumen acid-related modules. This pathway was defined as significantly enriched at *p* < 0.05. Coefficients of the Spearman correlation of acetic acid, propionic acid, and butyric acid with key metabolites were analyzed using the R-package Psych (2.0.7) and visualized using the R-package pheatmap (1.0.12). This correlation was statistically significant (*p* < 0.05).

### 2.6 Statistical analysis

Data on the production performance and rumen fermentation parameters were analyzed using one-way ANOVA and Tukey’s honest significant difference test using SPSS statistical software (version 20.0; SPSS Inc., Chicago, IL, USA). Statistical significance was set at *p* < 0.05. The results are expressed as the mean ± standard deviation. The experimental units were replicates, and the statistical model used was as follows:

$Y_{ij}=\mu +A_{i}+e_{ij}$

where *Y*_ij_ represents an observation, *μ* is the overall mean, *A*_i_ represents the effect of YC, and *e*_ij_ represents random error.

## 3 Results

### 3.1 Milk yield

The effects of YC on the average daily 4% FCM yield of Laoshan dairy goats are presented in Table 2. At weeks 4, 5, and 10, the average daily 4% FCM yield of YC1 was significantly greater than that of CON, YC2, and YC3 (*p* < 0.05). At week 4, the average daily 4% FCM yield of YC1 surpassed that of CON, YC2, and YC3 by 12.94, 14.29, and 9.09%, respectively; at week 5, it reached 16.77, 17.47, and 10.80%, respectively; and at week 10, it further rose to 24.55, 26.06, and 18.18%, respectively. At the 9th week, the average daily 4% FCM yield of YC1 was significantly greater than that of CON and YC2 (*p* < 0.05), with increases of 23.84 and 18.99%, respectively. However, no significant differences were observed between the YC1 and YC3 groups (*p* > 0.05).

**Table 2:** Effects of yeast culture on the milk yield of Laoshan dairy goats, kg/d.

Items^1^	CON	YC1	YC2	YC3	*p*-value
1st week	1.75 ± 0.17	1.78 ± 0.06	1.76 ± 0.03	1.83 ± 0.09	0.76
2nd week	1.76 ± 0.17	1.73 ± 0.12	1.71 ± 0.14	1.82 ± 0.17	0.81
3rd week	1.87 ± 0.31	1.78 ± 0.09	1.69 ± 0.13	1.88 ± 0.12	0.56
4th week	1.70 ± 0.04^b^	1.92 ± 0.10^a^	1.68 ± 0.59^b^	1.76 ± 0.12^b^	<0.05
5th week	1.67 ± 0.15^b^	1.95 ± 0.03^a^	1.66 ± 0.07^b^	1.76 ± 0.04^b^	<0.05
6th week	1.77 ± 0.90	1.95 ± 0.11	1.76 ± 0.11	1.83 ± 0.20	0.21
7th week	1.74 ± 0.07	2.07 ± 0.27	1.74 ± 0.11	1.85 ± 0.20	0.12
8th week	1.74 ± 0.08	2.18 ± 0.30	1.84 ± 0.11	1.90 ± 0.86	0.07
9th week	1.72 ± 0.13^b^	2.13 ± 0.23^a^	1.79 ± 0.08^b^	1.89 ± 0.20^ab^	<0.05
10th week	1.67 ± 0.16^b^	2.08 ± 0.17^a^	1.65 ± 0.11^b^	1.76 ± 0.69^b^	<0.05

^a,b^Values within the same row with different superscripts are significantly different (*p* < 0.05).

1 CON, control diet; YC1, CON + 10 g YC/d/goat; YC2, CON + 25 g YC/d/goat; YC3, CON + 40 g YC/d/goat; YC; yeast culture.

### 3.2 Rumen fermentation

The effects of YC on ruminal fermentation parameters in Laoshan dairy goats are shown in Table 3. Significant differences in ruminal pH were not observed between the CON, YC1, YC2, and YC3 groups (*p* > 0.05). Compared with the CON group, a significant decrease in ruminal NH_3_-N content was observed in the YC2 group (*p* < 0.05). However, significant differences were not observed in ruminal NH_3_-N content between the YC1, YC2, and YC3 groups (*p* > 0.05). Compared with the CON group, significant increases in ruminal acetic acid, butyric acid, and TVFA contents were observed among the YC1, YC2, and YC3 groups (*p* < 0.05), whereas no significant differences in ruminal acetic acid, butyric acid, and TVFA contents were observed between the YC1, YC2, and YC3 groups (*p* > 0.05).

**Table 3:** Effects of yeast culture on the rumen fermentation parameters of Laoshan dairy goats.

Items^1^	CON	YC1	YC2	YC3	*p*-value
pH	5.90 ± 0.18	5.89 ± 0.22	6.03 ± 0.16	6.22 ± 0.35	0.22
NH_3_-N, mg/dL	13.72 ± 2.42^a^	11.01 ± 1.97^ab^	9.28 ± 1.24^b^	11.04 ± 0.44^ab^	<0.05
Acetic, mmol/L	36.83 ± 6.09^b^	51.94 ± 6.82^a^	50.39 ± 6.57^a^	52.67 ± 11.33^a^	<0.05
Propionate, mmol/L	16.21 ± 2.11	22.05 ± 3.67	21.21 ± 2.90	21.33 ± 3.51	0.07
Butyrate, mmol/L	11.33 ± 1.21^b^	16.85 ± 3.48^a^	15.11 ± 1.94^a^	16.48 ± 2.14^a^	<0.05
TVFA, mmol/L	64.39 ± 9.35^b^	90.84 ± 13.92^a^	86.72 ± 11.25^a^	90.48 ± 16.96^a^	<0.05
Acetic/Propionate	2.26 ± 0.13	2.37 ± 0.08	2.38 ± 0.13	2.45 ± 0.13	0.21

^a,b^Values within the same row with different superscripts are significantly different (*p* < 0.05).

1 CON, control diet; YC1, CON + 10 g YC/d/goat; YC2, CON + 25 g YC/d/goat; YC3, CON + 40 g YC/d/goat; YC, yeast culture; NH_3_-N, ammonia-N; TVFA, total volatile fatty acids.

### 3.3 Characteristic analysis of OTUs

**Figure 1:**
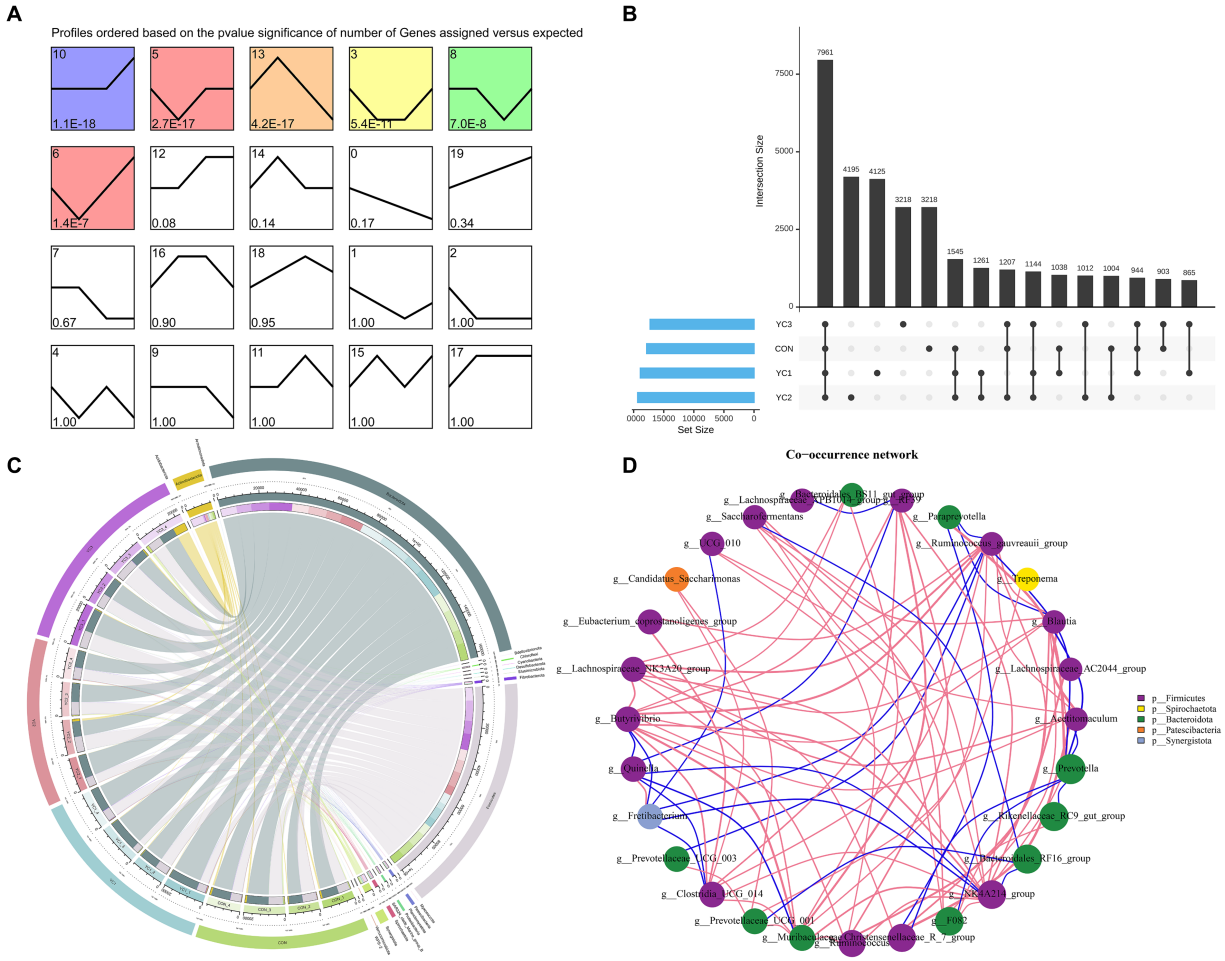
Characteristic analysis of operational taxonomic units (OTUs). **(A)** Temporal expression cluster analysis of OTUs. The color module showed a significant enrichment trend (*p* < 0.05). **(B)** Upset map of rumen fluid OTUs. The left bar chart shows the total elements in each original data set; vertical lines connect points to show intersections between data sets, and values represent common OTUs. **(C)** Circos map of OTUs in rumen fluid samples. The outermost circle on the left is sample grouping, on the right is phylum species, and the innermost part is the relative abundance percentage circle. The lines indicate the species and relative abundance information in the samples. **(D)** Change in the relative abundance of the genus. Using the Spearman test method, the top 20 genera in samples were selected for correlation analysis, and corresponding phyla were used as the legend. Results with *p* values greater than 0.05 were filtered out. The size of the point represents abundance, the thickness of the line represents correlation, and the color of the point represents the phylum. Red lines indicate positive correlations and blue lines indicate negative correlations.

A total of 1,734,238 tags were generated from 16 samples (with an average of 106,831 tags). These samples were obtained by randomly selecting four samples from each treatment group. After performing quality control, denoising, splicing, and de-chimerism, and eliminating singleton OTUs, 16 samples (average 96,824) generated 1,564,718 high-quality data points (valid tags). At a 97% similarity level, a total of 33,640 OTUs were described. The abundance of 33,640 OTUs was analyzed using STEM software. Six significantly enriched modules were identified (Figure 1A). Upset diagrams (Figure 1B) depict the number of OTUs that are unique to each group or shared among multiple groups. The CON, YC1, YC2, and YC3 groups had 17,820, 18,883, 19,329, and 17,254 unique OTUs, respectively, with 7,961 OTUs in total.

### 3.4 Abundance of ruminal bacteria

**Figure 2:**
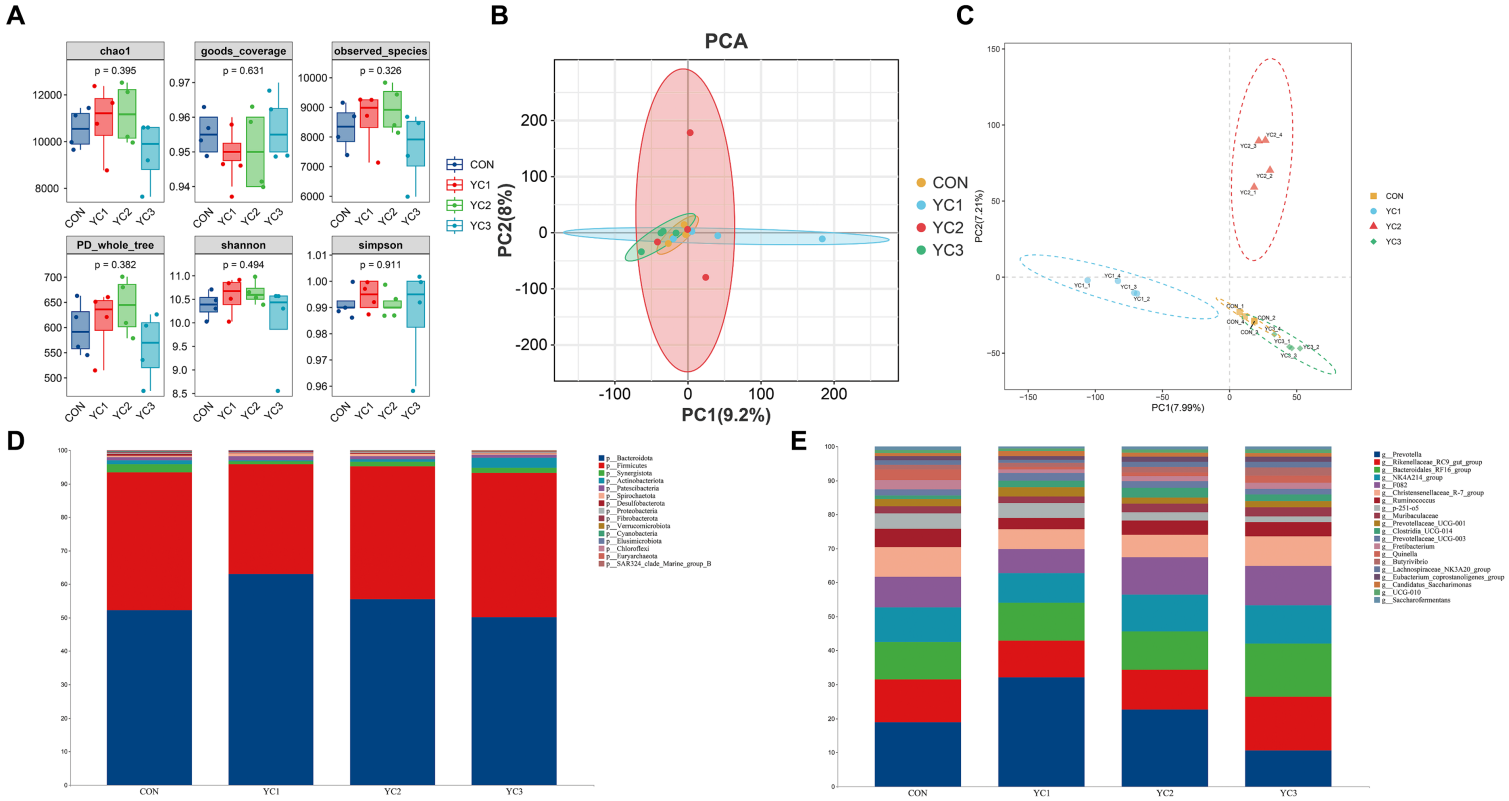
Abundance of ruminal bacteria. **(A)** Alpha diversity of rumen fluid microbiota of dairy goats with different treatments. The horizontal bar in the box represents the average. The top and bottom of the box represent the upper and lower quartiles, respectively. Single asterisk (*) means *p* < 0.05, and no asterisk means *p* > 0.05. **(B)** Principal component analysis (PCA) and **(C)** Partial least squares discriminant analysis (PLS-DA) models. Different colors represent samples from different groups of rumen liquid from dairy goats. The distance between the points on the map represents the similarity of all samples in terms of microflora composition and abundance. **(D)** Sankey diagram of species composition at the phylum level. Different colors represent different phyla. **(E)** Sankey diagrams of species composition at the genus level. Different colors represent different genera.

The α diversity of microbiota in the rumen fluid of dairy goats was analyzed by calculating the Chao1, Simpson, Shannon, Observed_species, PD_whole_tree, and Goods_coverage indexes. The results showed that the six α diversity indexes did not significantly differ among the different groups (*p* > 0.05) (Figure 2A). β diversity analyses were then performed, and the results showed that there were significant differences in phylogenetic distance among the four groups. Principal component analysis (PCA) showed that CON, YC1, YC2, and YC3 could not be distinguished, indicating that different treatments did not change the microbial diversity of the rumen fluid of dairy goats as a whole (Figure 2B). However, the partial least squares discriminant analysis (PLS-DA) model showed significant differences among CON, YC1, YC2, and YC3 (Figure 2C).

Changes in the composition of different microorganisms were analyzed at the phylum and genus levels. The results showed that Bacteroidota and Firmicutes were the two main phyla observed, and in CON, YC1, YC2, and YC3. Compared to that in CON, YC1 and YC2 showed relative increases in the abundance of Bacteroidota but decreases in the abundance of Firmicutes (Figure 2D). At the genus level, compared with the CON group, the relative abundance of *Prevotella* increased in the YC1 and YC2 groups but decreased in YC3, whereas the relative abundance of *Rikenellaceae_RC9_gut_group* increased in YC3. The relative abundances of *NK4A214_group* and *F082* were stable (Figure 2E). In summary, YC supplementation increased the abundance of the phylum Bacteroidota and genera *Prevotella* and *Rikenellaceae_RC9_gut_group* in the rumen of Laoshan dairy goats. Microbial co-occurrence networks have been widely used to explore the relationships in microbial communities. The Circos species relationship diagram at the phylum level of microbial community composition (Figure 1C) revealed that the CON, YC1, YC2, and YC3 groups mainly contained Bacteroidota and Firmicutes. An analysis of changes in relative abundance revealed that the genera *Prevotella*, *Rikenellaceae_RC9_gut_group*, *Bacteroi-dales_RF16_group*, *F082*, *p_251_o5*, *Muribaculaceae*, and *Prevotellaceae_UCG_001* of the phylum Bacteroidota and genera *NK4A214_group*, *Christensenellaceae_R_7_group*, and *Ruminococcus* of the phylum Firmicutes were highly abundant (Figure 1D). As shown in Figure 1D, the red lines between phyla represent positive correlations. Notably, the network demonstrated a multitude of positive correlations, as indicated by the dense web of red edges, indicating the existence of synergistic interactions among the phyla Firmicutes, Spirochaetota, Bacteroidota, Patescibacteria, and Synergistota.

### 3.5 Rumen fluid metabolic spectrum

**Figure 3:**
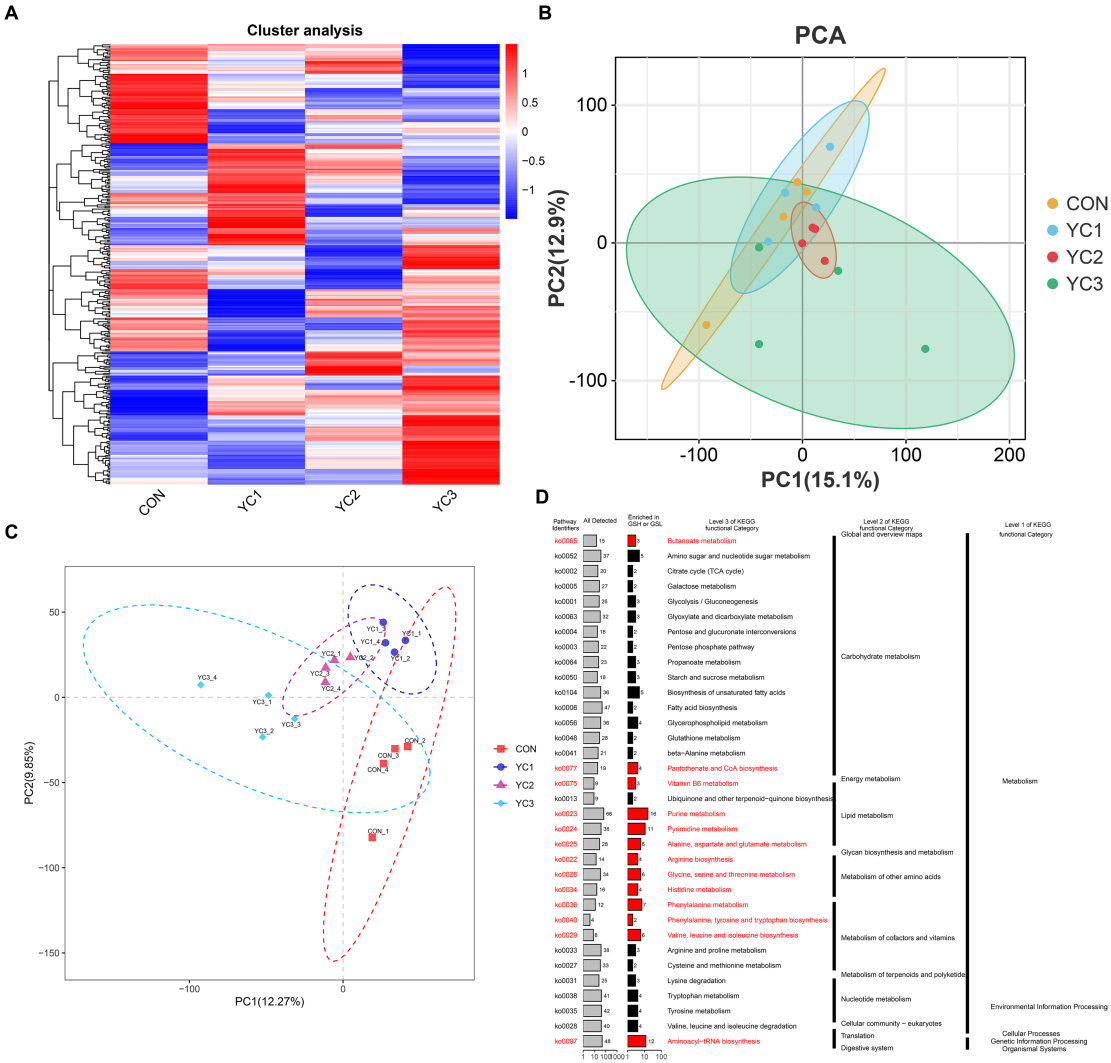
Rumen fluid metabolic spectrum. **(A)** Cluster analysis. Metabolite changes from green to red are shown for different samples. Darker red indicates greater abundance, darker green indicates lower abundance. **(B)** PCA and **(C)** PLS-DA diagrams. Different colors represent different groups of rumen fluid samples from dairy goats. The distance between points represents the similarity in microflora composition and abundance. **(D)** Kyoto encyclopedia of genes and genomes (KEGG) enrichment analysis, showing primary and tertiary pathways and KOID for each metabolic pathway. “All detected” indicates the number of metabolites annotated by KEGG data; “enrichment” indicates the number of differential metabolites enriched in the rumen acid pathway.

In this study, a UPLC-MS/MS non-targeted metabolomics analysis method was used to study the rumen fluid samples from dairy goats subjected to different treatments. Based on the results of secondary quality determination, 366 metabolites were annotated, of which 224 were annotated based on the HMDB database,^3^ and 203 were annotated based on the Kyoto Encyclopedia of Genes and Genomes (KEGG) database. A heat map of the expression profiles of the 366 metabolites was generated (Figure 3A). The PCA and PLS-DA maps were consistent with the results for the microorganisms (Figures 3B,C). The KEGG enrichment analysis of 336 metabolites showed that the metabolites were mainly concentrated in the primary metabolic pathways. The pathways with significant enrichment were purine metabolism; valine, leucine, and isoleucine bio-synthesis; phenylalanine metabolism; pyrimidine metabolism; aminoacyl-tRNA biosynthesis; alanine, aspartate, and glutamate metabolism; arginine biosynthesis; histidine metabolism; vitamin B6 metabolism; glycine, serine, and threonine metabolism; phenyl-alanine, tyrosine, and tryptophan biosynthesis; and pantothenate and CoA biosynthesis (Figure 3D).

### 3.6 Weighted co-expression network analysis of metabolomics and rumen fermentation parameters

**Figure 4:**
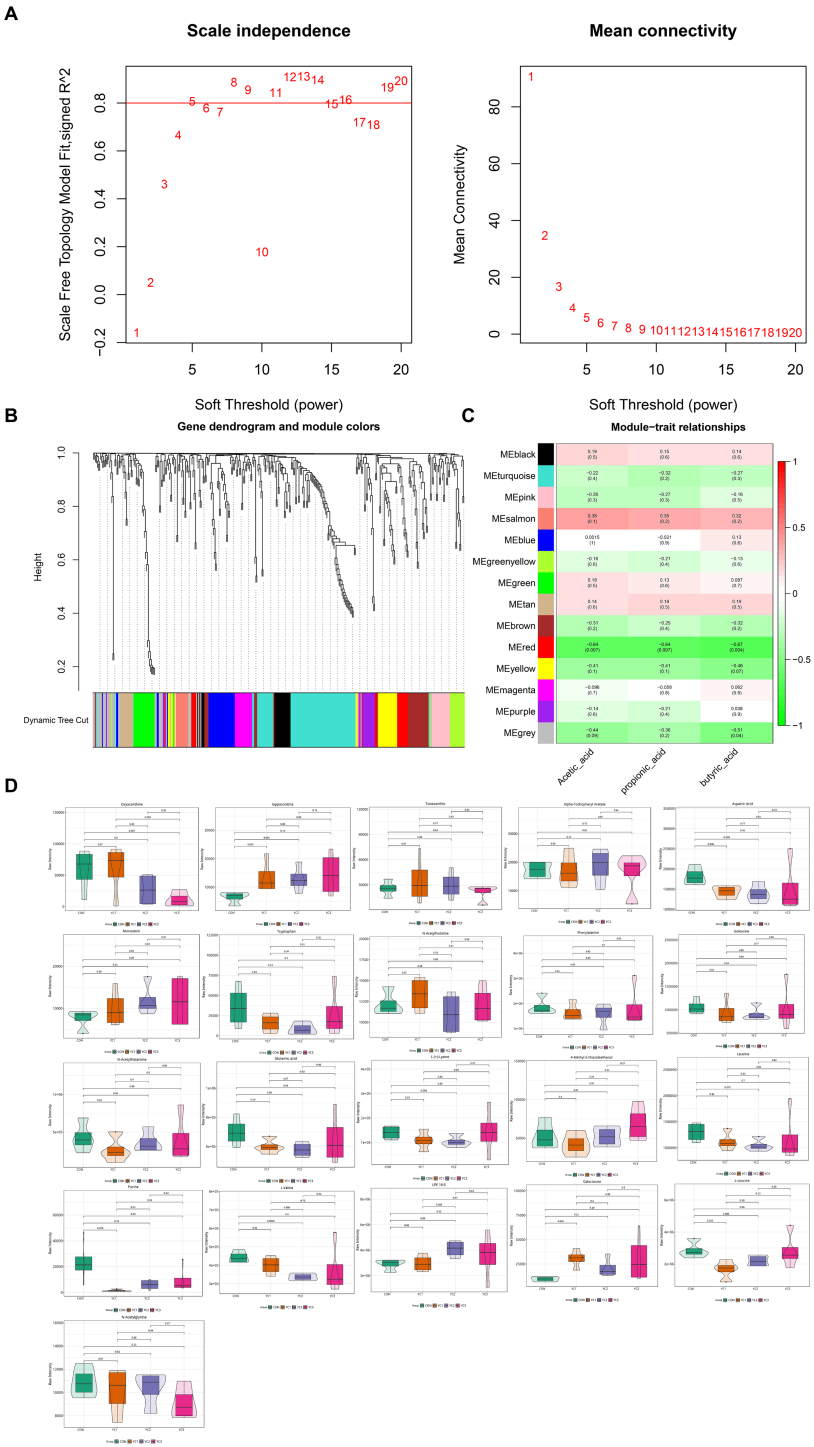
Weighted co-expression network analysis (WGCNA) of metabonomic and rumen fermentation parameters. **(A)** Determination of the soft threshold for WGCNA analysis. The scale-free fitting index and average connectivity show that a soft threshold greater than 5 satisfies a scale-free topology greater than 0.8. **(B)** Clustering tree map of different metabolites based on topological overlap. The dynamic cutting method identifies modules, shown in different colors below the tree view. **(C)** Heatmap diagram visualization module-feature association. Each row is a module characteristic, and each column is a feature, with correlation and *p* value in each unit. Red and green represent positive and negative correlations, respectively, with darker colors indicating stronger correlations. **(D)** Violin diagram of the abundance of 21 metabolites. *p* < 0.05 indicates a significant difference.

To explore the relationship between metabolites and rumen acid indexes, 366 metabolites were analyzed using WGCNA, and highly related metabolite modules were identified and correlated with the acetic acid, propionic acid, and butyric acid contents. WGC-NA used a soft threshold (power) of 5 to cluster 366 metabolites into 14 modules (Figures 4A,B). Among the 14 modules, 21 metabolites in the red module were significantly correlated with acetic, propionic, and butyric acids (*p* < 0.05, |*r*| > 0.5) (Figure 4C). The red module was negatively correlated with acetic, propionic, and butyric acids, indicating that the metabolites in this module may be the substrates used in the synthesis of acetic, propionic, and butyric acids. The abundances of 21 metabolites in the red module that were significantly related to rumen fluid were plotted. The results showed that the aspartic acid and L-leucine levels in the YC1 group were significantly lower than those in the CON group (Figure 4D).

### 3.7 Pathway enrichment analysis of important modules

**Figure 5:**
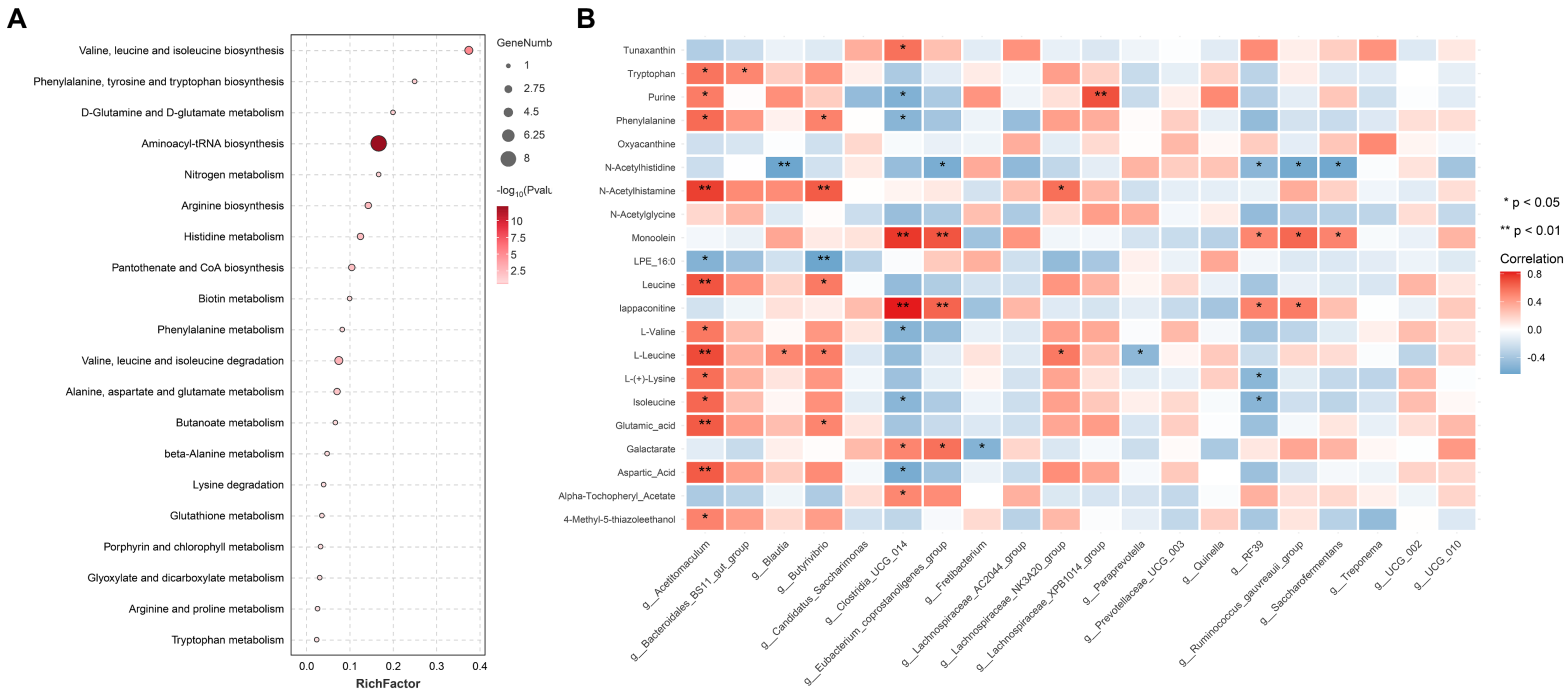
Pathway enrichment analysis of important modules. **(A)** KEGG enrichment analysis of differential metabolites significantly related to rumen acid. The *x*-axis shows the rich factor of each pathway, the *y*-axis shows the pathway names, and the color of the dot is the *p* value. A redder color indicates more significant enrichment. The size of the point represents the number of differential metabolites enriched. **(B)** Correlation analysis between rumen fluid-related metabolites and top 20 most abundant bacteria. Blue represents a negative correlation, red represents a positive correlation, and a darker color indicates a more significant correlation. An asterisk (^*^) indicates significance at *p* < 0.05, and no asterisk indicates *p* > 0.05.

Twenty metabolic pathways were identified, among which five were significant (*p* < 0.05) (Figure 5A): aminoacyl-tRNA biosynthesis; valine, leucine, and isoleucine biosynthesis; valine, leucine, and isoleucine degradation; arginine biosynthesis; and histidine metabolism. To further explore the potential correlation between these metabolites and related bacteria, R-package Psych software was used to calculate the Spearman correlation coefficients. Figure 5B shows the relationship between the key metabolites and bacteria. The results revealed a significant positive correlation between *Acetitomaculum* and aspartic acid (*p* < 0.05), a significant negative correlation between *Clostridia_UCG-014* and aspartic acid (*p* < 0.05), a significant positive correlation between *Acetitomaculum*, *Blautia*, *Lachno-spiraceae_NK3A20_group*, and *Butyrivibrio* and L-leucine (*p* < 0.05); and a significant negative correlation between *Paraprevotella* and L-leucine (*p* < 0.05). As shown in Table 4, the abundance of *Acetitomaculum* in the YC1, YC2, and YC3 groups was significantly greater than that in the CON group (*p* < 0.05); the abundance of *Clostridia_UCG-014* in the YC3 group was significantly greater than that in the CON group (*p* < 0.05); and the abundance of *Blautia* and *Paraprevotella* in the YC1 and YC2 groups was significantly greater than that in the CON group (*p* < 0.05).

**Table 4:** Differences in abundance of bacteria related to aspartic acid and L-leucine.

Items^1^	CON	YC1	YC2	YC3	*p*-value
Acetitomaculum	1.32 ± 0.03^c^	1.58 ± 0.07^a^	1.48 ± 0.04^b^	1.43 ± 0.05^b^	<0.01
Clostridia_UCG-014	2.22 ± 0.16^b^	2.42 ± 0.03^b^	2.33 ± 0.05^b^	3.01 ± 0.14^a^	<0.01
Blautia	1.32 ± 0.09^b^	1.63 ± 0.11^a^	1.57 ± 0.06^a^	1.36 ± 0.11^b^	0.01
Lachnospiraceae_NK3A20_group	2.65 ± 0.55	2.06 ± 0.91	3.06 ± 0.97	3.02 ± 0.85	0.47
Butyrivibrio	2.77 ± 0.70	2.76 ± 0.83	3.50 ± 0.99	4.83 ± 0.83	0.06
Paraprevotella	1.42 ± 0.01^b^	1.86 ± 0.09^a^	1.84 ± 0.01^a^	1.07 ± 0.04^c^	<0.01

^a,b^Values within the same row with different superscripts are significantly different (*p* < 0.05).

1 CON, control diet; YC1, CON + 10 g YC/d/goat; YC2, CON + 25 g YC/d/goat; YC3, CON + 25 g YC/d/goat; YC; yeast culture.

## 4 Discussion

The findings showed that dietary supplementation with YC has positive and significant effects on the milk yield of Laoshan dairy goats, which is consistent with the results of Khan et al. (28), who studied the effects of yeast supplementation on Beetal goats during early lactation and concluded that dietary yeast supplementation had beneficial effects on the milk yield. Moreover, the finding is consistent with that of Zaworski et al. (29), Dias et al. (4, 5), Nocek et al. (30), Halfen et al. (31), and Shi et al. (32) for cows and Baiomy (33) and Zicarelli et al. (34) for goats. In this study, the increased milk yield of dairy goats owing to YC supplementation was attributed to elevated levels of acetate, propionate, butyrate, and TVFAs in the rumen, which enhanced the fermentation activity of cellulolytic bacteria (35). In contrast, our findings are inconsistent with the results of Hadjipanayiotou et al. (36), who evaluated the effect of YC on milk yield in Damascus goats and reported that the milk yield did not differ between animals. The variance in results might be attributed to differences in the yeast strains and animal species.

Dietary carbohydrates are fermented by ruminal bacteria, fungi, and protozoa into end products, including VFA (e.g., acetate, propionate, and butyrate), which constitute nearly 50% of the energy requirements for ruminants (37). In this study, dietary YC supplementation increased the ruminal acetic, butyrate, and TVFA contents. These findings are consistent with those of Carpinelli et al. (38), Zhu et al. (14), Sun et al. (39) for cows, and Xue et al. (40), Özsoy et al. (41), and Ogbuewu et al. (42) for goats. In our study, we observed that YC supplementation increased the abundance of fiber degrading bacteria (Bacteroidetes and *Prevotella*) in the goat rumen, thereby promoting cellulose metabolism, which ultimately led to an increase in the ruminal acetate, butyrate, and TVFA contents (43). Studies have shown that YC supplementation reduces NH_3_-N levels in ruminants. For instance, a study on dairy cows observed a tendency for lower rumen NH_3_-N concentrations with YC compared to the control (44). In this study, among the 21 metabolites that presented significant correlations with rumen fermentation parameters, aspartic acid and L-leucine contents in the rumen of the YC group were significantly reduced. Furthermore, KEGG analysis of these 21 metabolites highlighted significant amino acid synthesis pathways, including aminoacyl-tRNA biosynthesis, valine, leucine, arginine biosynthesis, and histidine metabolism. These results suggest that YC supplementation enhances the efficiency of microbial protein synthesis in the rumen, thereby enabling more effective utilization of NH_3_-N (45).

Higher microbial diversity in the mammalian gastrointestinal system is often associated with a stronger metabolic capacity (46). In this study, β-diversity analyses showed that microbial diversity was increased in the YC groups. Furthermore, studies have shown that feed efficiency in dairy cows was significantly associated with lower microbial diversity in the rumen (47). However, YC supplementation increased the abundance of the phylum Bacteroidota and genera *Prevotella* and *Rikenellaceae_RC9_gut_group* in the rumen of goats, mitigating the negative impacts on milk production and feed efficiency. The rumen is a complex microbial anaerobic fermentation chamber that harbors one of the most diverse intestinal microbial communities in the animal kingdom (48). Firmicutes and Bacteroidota are the dominant species in the goat rumen (49). The positive correlations observed among the phyla Firmicutes, Spirochaetota, Bacteroidota, Patescibacteria, and Synergistetes in this study are consistent with the synergistic interactions among related taxa identified in previous studies (50). These relationships may arise from shared metabolic pathways or mutualistic interactions, such as cross-feeding, in which one genus produces a metabolite utilized by another (51). Moreover, the relative abundance of Bacteroidetes increased in the rumen of the dairy goats treated with YC in this study, which is similar to the findings of Li et al. (52) regarding cows. The positive effects of YC on *Prevotella* growth have also been previously documented (53) and supported by the results of the current study. Such effects are related to YC growth factors (i.e., organic acids, B vitamins, and AA) that stimulate fiber-digesting bacteria, such as Bacteroidetes and *Prevotella* (54, 55).

The metabolites in the rumen mainly include nutrients that can be used by the host and rumen microorganisms, and differences in the levels of ruminal metabolites are associated with changes in the microbiota (56). We identified 13 tertiary metabolic pathways, which primarily included secondary metabolic pathways of lipid metabolism, glycan biosynthesis and metabolism, amino acid metabolism, and cofactor and vitamin metabolism. These findings are consistent with the results of Li et al. (57). As the main pathways of microbial VFA production, lipid metabolism, glycan biosynthesis and metabolism, and carbohydrate metabolism play important roles in the rumen (3, 58, 59). In addition, microorganisms satisfy the host’s nutritional needs by performing amino acid metabolism and cofactor and vitamin metabolism to produce amino acids, vitamins, and cofactors (60). This study observed the consistency between the differences in metabolite types between the YC and CON groups and the differences in microbial populations, indicating that the diversity of microbial populations also affects the diversity of rumen metabolite types. Similarly, Xue et al. (61) also reached similar conclusions in a study on the impact of different feed types on the rumen microbiome and serum metabolome in lambs.

Goat rumen metabolites were significantly negatively correlated with acetate, butyrate, and propionate, which is consistent with a previous study in which negative correlations were observed between specific metabolites and rumen fermentation parameters (62). This study found that among the 21 metabolites negatively correlated with ruminal acetate, propionate, and butyrate, YC supplementation significantly reduced the ruminal aspartic acid and L-leucine. This observation is in line with a previous study in which YC significantly influence the metabolite profiles in the rumen of dairy cows, thereby affecting the fermentation processes (63). Studies have also shown that L-leucine could be converted into branched chain VFAs, such as acetic acid and butyric acid, during oxidative deamination (64). In addition, Jalc and Ceresnáková (65) investigated the effect of aspartic acid on rumen fermentation and found that aspartate influenced propionate production during rumen fermentation. To explore the microbes that caused the differences in ruminal aspartic acid and L-leucine levels, this study conducted a correlation analysis between aspartic acid and L-leucine and the top 20 most abundant genera in the rumen, and then it performed an analysis of variance among the groups for the correlated microbial populations. The negative correlation between aspartic acid and L-leucine and *Clostrid-ia_UCG-014* and *Paraprevotella* and the greater abundance of *Clostridia_UCG-014* and *Paraprevotella* in the YC groups revealed that differences in these genera in the rumen lead to the differences in ruminal aspartic acid and L-leucine levels.

Further investigation into these specific metabolites could reveal more about their roles and how they influence the rumen’s acidic environment. Among the five significant pathways identified in this study, aspartic acid directly participates in the amino-acyl-tRNA biosynthesis and arginine biosynthesis pathways and L-leucine directly participates in the aminoacyl-tRNA biosynthesis, valine, leucine, and isoleucine biosynthesis, and valine, leucine, and isoleucine degradation pathways. Moreover, research has shown that aminoacyl-tRNA biosynthesis is the foundation of microbial protein synthesis in the rumen, supporting their growth and metabolism, which in turn influences the fermentation process in the rumen (66). In addition, microorganisms can use L-leucine to produce propionyl-CoA and acetyl-CoA through valine, leucine, and isoleucine degradation metabolic pathways, and then propionyl-CoA and acetyl-CoA can be further metabolized into VFAs, such as propionic acid and acetate, thus affecting rumen fermentation parameters (67). Integrating the findings of this study with previous research reveals that the impact of YC on ruminal fermentation parameters in goats was attributed to its promotional effect on *Clostridia_UCG-014* and *Paraprevotella*, which then utilize more aspartic acid and L-leucine through pathways such as aminoacyl-tRNA biosynthesis and valine, leucine, and isoleucine degradation. These changes ultimately lead to alterations in ruminal fer-mentation parameters.

## 5 Conclusion

Yeast culture dietary supplementation at 10 g/day per goat improved the milk yield and ruminal fermentation parameters in Laoshan dairy goats. Moreover, YC increased the ruminal *Clostridia_UCG-014* and *Paraprevotella* abundance, which facilitated aspartic acid and L-leucine utilization by these genera, thereby enhancing the ruminal acetic, butyrate, and TVFA contents and reducing the ruminal NH_3_-N content.

## Data availability statement

The original contributions presented in the study are included in the article/supplementary material, further inquiries can be directed to the corresponding author.

## Ethics statement

The animal study was approved by the Animal Administration and Ethics Committee of Qingdao Agricultural University, Animal Science and Technology College. The study was conducted in accordance with the local legislation and institutional requirements.

## Author contributions

ZL: Writing – original draft, Writing – review & editing. YH: Data curation, Formal analysis, Writing – review & editing. HL: Investigation, Writing – review & editing. YL: Funding acquisition, Project administration, Resources, Writing – review & editing. MC: Supervision, Writing – review & editing. FZ: Methodology, Writing – review & editing. YG: Methodology, Project administration, Writing – review & editing.

## Footnotes

^1^ www.metaboanalyst.ca/

^2^ https://smpdb.ca/

^3^ https://hmdb.ca/metabolites
